# What do we understand from clinical and mechanistic studies on acupuncture treatment for hypertension?

**DOI:** 10.1186/s13020-015-0070-9

**Published:** 2015-11-30

**Authors:** Ling Cheng, Peng Li, Stephanie Cheeyee Tjen-A-Looi, John Charles Longhurst

**Affiliations:** Department of Acupuncture, East Hospital, Shanghai, China; Susan-Samueli Center for Integrative Medicine, School of Medicine, University of California, Irvine, USA

## Abstract

The outcome of acupuncture on hypertension treatment is inconclusive. This study aims to evaluate the influence of acupuncture on hypertension, based on findings from mechanistic studies over the course of decades particularly those conducted at the University of California, Irvine. Low-current and low-frequency electroacupuncture (EA) at P5–6 (overlying the median nerve) and S36–37 (overlying the deep peroneal nerve) reduced high blood pressure in a subset of patients (~70 %) with mild-to-moderate hypertension, in a slow-onset (4–8 weeks) but long-lasting (1–2 months) manner. EA inhibited cardiovascular sympathoexcitatory neurons through activation of neurons in the arcuate nucleus of the hypothalamus, the ventrolateral periaqueductal gray in the midbrain and the nucleus raphe pallidus in the medulla, through inhibiting the activity of premotor sympathetic neurons in the rostral ventrolateral medulla (rVLM). Several neurotransmitters such as glutamate, acetylcholine, opioids, GABA, nociceptin, serotonin and endocannabinoids were involved in this EA-induced hypotensive response. The long-lasting inhibition of hypertension induced by EA was related to opioids and GABA in the rVLM, neural circuitry between the arcuate and ventrolateral periaqueductal gray, and prolongation of the increase in preproenkephalin mRNA levels and enkephalin levels in the rVLM and arcuate. Moreover, the long-lasting inhibition of sympathetic activity by EA was confirmed in EA-treated hypertensive patients with decreased levels of norepinephrine, renin and aldosterone.

## Background

Hypertension is the most common chronic disorder, affecting approximately one billion individuals worldwide [1]. Nearly one-third of the US adult population is hypertensive, and the lifetime risk of developing hypertension approaches 90 % [2]. There are many antihypertensive medications, but these have adverse side effects. Drug therapy indiscriminately blocks many receptors that lead to a multiplicity of side effects [3, 4]. Consequently, there is growing interest in alternative medical treatments such as acupuncture, which may complement, and offer a potential alternative to, pharmacological therapy [5]. Despite the increasing worldwide interest in acupuncture [6, 7], many Western physicians are reluctant to recommend acupuncture, owing to its controversial mechanism of action in hypertension treatment and the unclear physiological mechanisms underlying its hypotensive effect [8].

A number of clinical reports have suggested that acupuncture reduces blood pressure (BP) [9–18]. However, in 2004, the Stop Hypertension with Acupuncture Research Program (SHARP) trial concluded that acupuncture could not decrease BP [19]. The acupuncture treatments used in that study included stimulation at many acupoints that have not been experimentally shown to be effective in reducing elevated BP in a standardized treatment group. Moreover, 24-h measurement of systolic and diastolic BPs with an ambulatory BP monitor was not performed. Recent reviews have concluded that acupuncture may lower BP in patients on antihypertensive medications, but that acupuncture alone does not decrease high BP [20]. Conversely, other clinical reports have shown that acupuncture can reduce BP [20–22].

There are many weaknesses in these prior clinical studies, including small sample sizes, insufficient randomization, lack of adequate control groups [23] and the use of *meridian* and *qi* hypotheses in place of modern scientific principles [3, 19]. Additionally, few studies monitored BP continuously (24-h BP monitoring) and, in most studies, the patients were on antihypertensive medications. In some studies, the period of follow-up after acupuncture treatment was not long enough [24]. Thus, rigorously and properly designed clinical trials to evaluate the influence of acupuncture on hypertension are required.

This commentary will focus on issues that should be considered in a rigorous and properly designed clinical study. (1) Does the study examine manual acupuncture or electroacupuncture (EA)? (2) Which effective and control acupoints should be used? (3) What stimulation parameters should be studied? (4) What is the best duration of treatment? (5) For how long should we perform follow up after treatment? These aspects of acupuncture are important issues in determining the efficacy of acupuncture. Our findings to date show a significant BP-lowering effect of acupuncture. We use standardized sets of acupuncture acupoints that have been shown to display specific underlying mechanisms to inhibit sympathetic activity and lower elevated BP [25–29]. By contrast, in studies that use many acupoints to treat symptoms related to hypertension-such as headache, dizziness, anxiety, constipation, insomnia, and lack of appetite, the outcomes and effects of treatment are more complicated. Therefore, efforts to treat hypertensive patients should be based on experimental laboratory findings.

### Acupoint specificity

Chinese medicine (CM) describes 12 bilateral and eight extra meridians that connect internal splanchnic organs to external regions on the extremities and body trunk in the theory of “*meridians* and *collaterals*”. This theory suggests that meridians and collaterals are pathways for *qi*. According to modern scientific concepts, the meridian serves an integrative function through the sensory and ultimately the autonomic nervous systems and may also influence the endocrine and immune systems in CM [25, 30]. In modern medical concepts, the mode of action of acupuncture is influenced by central processing and integration of peripheral afferents originating from the splanchnic organs and somatic nerves (see below) [8, 25]. However, the mechanisms of action of acupuncture in the treatment of essential hypertension are not yet fully understood, and thus, require further investigation.

A number of scientists have doubted acupoint specificity because acupuncture releases opioids systemically, and these have the potential to exert widespread actions. For example, acupoint specificity was shown with respect to stimulation of the visual cortex [31]. However, this publication was later withdrawn [32] because the authors could not confirm acupuncture point specificity with regard to its analgesic effect. To explore this concept, we examined the effectiveness of activating different acupuncture points on reflex-induced increases in BP by performing stimulation of visceral nerves in cats [33]. Stimulation of visceral sensory fibers activated sympathoexcitatory cardiovascular premotor sympathetic neurons in the rostral ventrolateral medulla (rVLM) resulting in an increase in BP. We also evaluated acupoint specificity by performing extracellular recordings of neurons in the hypothalamus. Stimulation of various acupoints differentially activated arcuate neurons [32]. The neurons in the arcuate receive more inputs from nerves underlying acupoints at P5–6 (*Neiguan*-*Jianshi*, overlying the median nerve), S36–37 (*Zusanli*-*Shangjuxu*, overlying the deep peroneal nerve), LI4–11 (*Hegu*-*Quchi*, overlying the branch of median nerve and deep radial nerve), H5–7 (*Tongli*-*Shenmen*, overlying the ulnar nerve), and S2–G2 (*Sibai*-*Tinghui*, overlying the cranial nerves: the branch of the trigeminal nerve and the branch of the facial nerve) than they do from nerves underlying acupoints at LI6–7 (*Pianli*-*Wenliu*, overlying the superficial radial nerve) and G37–39 (*Guanming*-*Xuanzhong*, overlying the superficial peroneal nerve). Conversely, we observed that EA at LI6–7 (*Pianli*-*Wenliu*) and K1-B 67 (*Yongquan*-*Zhiyin*, superficial medial plantar and digital nerves) does not influence elevated BP. However, EA at P5–6, S36–37, and LI10–11 (*Shousanli*-*Quchi*, overlying the deep radial nerve) was most effective in its influence on cardiovascular function [8, 34]. EA at these acupoints and direct stimulation of deep or cutaneous nerves underneath these acupoints also produced similar point-specific results in models of gallbladder-evoked increased BP [26]. Therefore, we treated hypertensive patients with acupuncture at P5–6 + S36–37 as effective acupoints and at LI6–7 + G37–39 as control acupoints.

### Manual acupuncture and electroacupuncture

In the rat model of reflex-induced hypertension, EA and manual acupuncture (MA) matched for frequency induced similar cardiovascular responses [28, 29]. We have shown that EA at P5–6, H5–7 (overlying the ulnar nerve), or S36–37 with low current (2 mA) and frequency (2 Hz) for 30 min inhibited the reflex increase in BP for 30–40 min. Manual acupuncture decreased elevated BP similarly, while sham acupuncture involving needle insertion without manipulation at P5–6 or LI6–7 acupoints did not attenuate gastric distension-induced increases in BP. Thus, sham acupuncture can serve as a control for EA studies [35, 36]. We use EA because it is a standardized treatment and induces less injury to the receptors and nerve endings underneath the acupoints.

### Stimulation parameters

Previous studies using compound action potential recording techniques have shown that finely myelinated fibers are the predominate afferents stimulated during low-current, low-frequency EA [25, 37]. However, using a single afferent recording technique, we showed that both myelinated and unmyelinated nerve fibers were activated during stimulation (at P5–6 acupoints) of the median nerve with very low current [26]. Of the 62 fibers recorded, 37 % were unmyelinated (C fibers ≤2.5 m/s) and 63 % were myelinated (Aδ fibers >2.5 m/s). We used motor threshold as a guide to achieve similar stimulus intensities between animals used to examine the effects of EA [26]. Thus, both myelinated and unmyelinated fibers likely participate in the cardiovascular responses to acupuncture.

We also examined the importance of C fibers during EA using neonatal capsaicin-treated rats [38] Depletion of substance P from unmyelinated afferent C-fibers reduced responsiveness to EA modulation of gastric distention-induced BP increases. Thus, inhibition of the cardiovascular excitatory reflex by EA at P5–6 requires, at least in part, input from unmyelinated C fibers.

We found that low-frequency and low-current EA optimally influenced reflex-induced hypertension [28, 29]. Increasing EA stimulation frequency to 40 or 100 Hz resulted in less, or even no, inhibition of the increase in BP. Moreover, 100-Hz stimulation induced minimal input to premotor sympathetic neurons in the rVLM. We observed a reciprocal relationship between the frequency of stimulation and EA-related afferent responses [28]. Therefore, when we treat hypertensive patients, we only use low-current, low-frequency EA.

### Stimulation duration

Usually, optimal acupuncture therapeutic effects can be achieved following an average of 30 min of stimulation [27, 39–41]. In animal experiments, the effect of EA was maintained for about 1 h after the end of EA application [8, 30, 31, 47, 67–69]. EA stimulation for 20–40 min activated long-loop pathways in the brain and induced the release of a number of neurotransmitters, including γ-aminobutyric acid (GABA), serotonin (5-hydroxytryptamine, 5-HT), acetylcholine, nociceptin, β-endorphin, enkephalins, and endocannabinoids in the hypothalamus, midbrain and medulla [8]. Thus, a single application of approximately 30 min of EA is necessary to achieve prolonged and optimal modulation of cardiovascular function.

However, some acupuncturists and researchers applied acupuncture for brief periods only, often <1 min, and observed decreases in BP and heart rate [29]. In this regard, short-term somatic nerve stimulation induced the somatosensory reflex that influences BP and heart rate [42]. The hemodynamic responses to these forms of stimulation operate via different mechanisms from those that underlie the effects of more prolonged stimulation. With brief stimulation, hemodynamic responses returned to normal within a few minutes [43]. To observe a clinical effect in hypertensive patients, we apply weekly 30-min EA for 8 weeks.

## Clinical studies

### Studies in healthy subjects

EA applied to healthy subjects at rest does not change BP or heart rate [40], and EA inhibited exercise stress-induced increases in systolic and mean BPs (SBP and MBP) as well as the double product (SBP × heart rate, an index of myocardial oxygen demand) [33] in ~70 % of healthy humans, without significant change of diastolic BP (DBP). In contrast, EA at control acupoints G37–39 did not significantly alter exercise stress-related increases in BP or the double product suggesting that EA at certain acupoints (e.g., P5–6) reduced sympathoexcitatory reflex-induced increases in BP.

### Effects of EA on hypertensive patients

The acupoints and parameters of EA that most effectively reduce SBP/DBP in patients with mild-to-moderate hypertension (BP 140–180/90–110 mmHg) were studied in a single-blinded randomized clinical trial [28]. A group of 35 hypertensive patients without medication were given EA at P5–6 and S36–37, once weekly for 30 min, for 8 weeks, and assessed with 24-h ambulatory BP monitoring. After 8 weeks of treatment, ~70 % of patients (responders) demonstrated significant reductions in peak and average SBP/24 h. DBP/24 h was also decreased. Heart rate was unchanged. After termination of EA treatment, SBP remained reduced for 4 weeks, but after 8 weeks returned to near pretreatment levels. About 30 % of patients did not respond to the EA treatment (non-responders). In 32 patients treated at control acupoints (LI6–7 + G37–39), neither SBP/24 h nor DBP/24 h were reduced. After 8 weeks of EA treatment, the changes in SBP (*P* = 0.031) and DBP (*P* = 0.031) were significantly different between the two groups (Student’s *t* test). This beneficial effect of EA was slow in onset but long-lasting [41].

Our recent data also showed that EA weekly for 8 weeks decreased the plasma levels of catecholamine, renin and aldosterone in hypertensive patients who responded to EA treatment [28]. These plasma hormones did not change in patients who did not respond well to the treatment [28].

The mechanism of action of acupuncture is mainly related to the integrative function of the central nervous system at specific points in reducing elevated BP; this function inhibits sympathetic activity and the renin–angiotensin–aldosterone system, and principally decreases SBP.

## Mechanisms of acupuncture: central nervous system and neurotransmitter studies

### The role of rVLM in the cardiovascular response to acupuncture

The rVLM is a critical source of premotor sympathetic neurons that regulates sympathetic outflow to the intermediolateral column of the spinal cord [41, 42]. The rVLM was found to be indispensable for EA-mediated inhibition of defense reaction-induced elevations in BP during stimulation of hypothalamic or midbrain defense areas using morphological (horseradish peroxidase) and electrophysiological techniques. Iontophoresis of morphine inhibited the evoked excitation of rVLM premotor sympathetic neurons, while naloxone reversed EA-related inhibition of the activated neurons. These authors’(Dr. Li’s group in Shanghai) data also suggested an opioid mechanism underlying the effects of acupuncture in the rVLM [44–46].

Acupuncture did not influence BP under normal physiological conditions, such as in normotensive subjects [34]. As such, the mechanisms underlying the effects of acupuncture were examined under conditions of elevated sympathetic activity, such as brief and sustained hypertension [47, 48]. Using in vivo experiments, various mechanisms of action of acupuncture were investigated revealing the following: (1) EA at select acupoints such as *Neiguan*-*Jianshi* along the pericardial meridian (P5–6) reduces sympathoexcitatory-related increases in BP [26, 49]; (2) EA induces a beneficial effect by reversing demand-induced myocardial ischemia through its reduction of myocardial oxygen demand [26]; (3) EA induces inhibition of evoked premotor sympathetic rVLM neuronal discharge [33, 34]; and (4) the long-lasting inhibitory effect of acupuncture persists during and after the application of EA for at least 1 h [50]. Microinjection or iontophoresis of naloxone or gabazine, an antagonist of GABA_A_ receptors, into the rVLM, reduced the prolonged EA-mediated inhibition of reflex-induced neuronal activity and BP increases [34, 50]. EA specifically inhibited elevated BP and rVLM activity through activation of µ and δ opioid receptors, but not κ opioid receptors in the rVLM [51]. We also showed that low-frequency and low-intensity EA induced c-Fos expression in enkephalinergic rVLM neurons [52].

We have examined point specificity in rVLM neurons characterized as cardiovascular and sympathoexcitatory. These rVLM neurons processed somatic input during acupuncture stimulation [33, 36, 45] at P5–6 and LI4–11 acupoints, along the *large intestine meridian*, and provided more convergent afferent inputs to cardiovascular premotor sympathetic neurons in the rVLM than did cardiovascular inactive points (LI6–7, G37–39) suggesting a point-specific integration in the central nervous system [33].

### Hypothalamic and midbrain nuclei in the acupuncture response

The hypothalamus and midbrain are involved in the action of EA in modulating elevated BP responses [46, 53]. The blockade of glutamatergic receptors in the arcuate nucleus reversed EA-induced inhibition of sympathoexcitatory responses, confirming the critical role of this nucleus in acupuncture [54, 55]. The neurons in the arcuate nucleus are rich in endorphins and project directly to the rVLM, suggesting they might activate µ-opioid receptors and consequently inhibited rVLM neurons [56]. The β-endorphinergic arcuate neurons might be involved in the inhibitory action of EA on cardiovascular excitatory responses [27, 55].

We also evaluated the role of the ventrolateral periaqueductal gray (vlPAG) and its interaction with the hypothalamic arcuate nucleus in EA-induced cardiovascular sympathoexcitatory responses [54–56] and whether excitatory projections from the arcuate to the vlPAG and vice versa are essential for EA inhibition of sympathoexcitatory cardiovascular BP responses.

The neurons in the arcuate and vlPAG with low spontaneous activity (4–5 impulse/s) responded to both visceral and acupoint-somatic afferent stimulation [55]. These neurons also responded to baroreceptor input and displayed frequency discharges correlated with the cardiac cycle, displaying strong cardiac rhythmicity. These neurons showed gradation of responses to stimulation of different acupoints, providing a cellular basis for point-specific responses. In this respect, evoked responses in both the arcuate and vlPAG were the greatest during stimulation of the P5–6, LI4–11, H5–7, and S2–G2 acupoints compared with the responses during stimulation of LI6–7 and G37–39. These neurons responsive to EA enhanced evoked activity in the arcuate nucleus and vlPAG.

Phenotypical enkephalinergic cells in the vlPAG, which are activated by EA, express c-Fos [52]. Endorphinergic arcuate nucleus projections combined with enkephalinergic vlPAG and reciprocal projections comprise, in part, the long-loop pathway that eventually inhibit rVLM neurons, and which is critical for the sympathoinhibition occurring during electroacupuncture [27, 57].

This long-loop pathway in EA inhibition involves a number of excitatory and inhibitory neurotransmitters. These neurotransmitters specific to cardiovascular nuclei include glutamate, acetylcholine in the arcuate nucleus, endocannabinoids and GABA (γ**-**aminobutyric acid) in the vlPAG, serotonin in the nucleus raphé obscurus and the nucleus raphé pallidus (NRP), and opioids and GABA in the rVLM, among others [35, 44, 53, 55, 56].

### Role of the spinal cord in the cardiovascular response to acupuncture

Reflex responses to activation of somatic and visceral afferents are integrated into cardiovascular supraspinal regions and the spinal cord. The spinal cord relays outputs from the central nervous system to effector organs involved in cardiovascular reflex regulation [58]. The dorsal horn of the spinal cord is a major center for EA-induced analgesia [59]. The number of c-Fos-immunoreactive neurons is increased by both low- and high-frequency EA at the *Zusanli* (S36) acupoint in the superficial laminae (I and II) in the dorsal horn of the spinal cord [59]. Moreover, opioid and nociceptin-like immunoreactivities are present in the spinal sympathetic nuclei (intermediolateral column, IML) [60, 61]. Therefore, we investigated the spinal mechanism of action of EA in sympathoexcitatory cardiovascular responses. Nociceptin and opioids in the dorsal horn and IML of the spinal cord reduced the response to rVLM-induced sympathoexcitation or visceral induced reflex responses suggesting that these neurotransmitters acted on sympathetic outflow [62]. This study also showed that nociceptin functions independently from opioids in mediating the effects of acupuncture.

Descending pathways from the brain stem influenced the segmental processing of somatic inputs during EA [61, 63]. Furthermore, stimulation of specific dermatomes with somatic nerve stimulation elicited excitatory and inhibitory responses in both IML and dorsal horn interneurons [64]. These interneurons link in the spinal cord circuitry underlying autonomic control [65]. Opioids and nociceptin could be involved in the activity of interneurons in the spinal cord during the EA response.

## Long-lasting effect of acupuncture

Thirty minutes of EA application induced a significant and immediate post-stimulation short-term reduction in DBP [66]. In a small study of 50 patients with essential hypertension, Chiu et al. [23] administered acupuncture for >30 min, both SBP and DBP were lowered by 10–20 mmHg.

In conscious animals (dogs and spontaneous hypertensive rats) EA modulated BP for 1–12 h [67–69]. EA-induced inhibition of the cardiovascular excitatory reflex response lasted between 1 and 6 h in anesthetized rabbits, rats, and cats [26, 49, 69, 70]. In anesthetized animals, EA inhibits sympathoexcitatory cardiovascular premotor rVLM neuronal activity for over 1 h after cessation of EA [34, 45]. The initial occlusive interaction of somatic and visceral convergent afferent input in rVLM is very short-lived, while prolonged stimulation of somatic afferents (30 min EA) leads to long-lasting inhibition of visceral-evoked neuronal responses. Thus, inhibition of cardiovascular function by acupuncture is based on 30 min of somatic afferent-evoked rVLM activity influencing central regulation of sympathoexcitatory responses [34].

### Neurotransmitter specificity

This prolonged inhibition effect of EA on rVLM sympathetic premotor neuronal responses to excitatory visceral input involves specific neurotransmitters. In particular, opioids and GABA but not nociceptin are important in the inhibition of rVLM sympathetic premotor neurons during this long-lasting EA effect [50].

Non-responsiveness to acupuncture treatment occurred in about 30 % of subjects, and an EA analgesic response occurred in about 70 % of patients [71, 72]. Similar acupuncture effectiveness in terms of sympathoexcitatory cardiovascular responses [40, 73] and effects on hypertension [41] were observed. We investigated the neurotransmitter that antagonizes the actions of opioids, and found that, within the rVLM, cholecystokinin 8 reversed the effects of EA, while blockade of its receptor converted an EA non-responsive subject to an EA responder [73]. Neurotransmitter mechanisms might be involved in the effectiveness of acupuncture.

### Long-loop pathway

The hypothalamus, midbrain and medulla participate in the prolonged action of acupuncture. Microinjection of kainic acid into the hypothalamic region blocked EA-mediated inhibition of reflex-induced hypertension [27]. Prolonged inhibition of rVLM neurons by EA requires an intact arcuate nucleus [27, 55]. Reciprocal excitatory projections between the arcuate nucleus and the vlPAG [74] prolonged EA-cardiovascular regulation for 30–60 min [8]. Meanwhile, projections from the vlPAG to the NRP and then the rVLM contributed to the serotonin-induced inhibition of rVLM neurons [57, 75, 76]. Inhibition of sympathoexcitation with acupuncture is long-lasting and involves the arcuate nucleus, vlPAG, NRP and rVLM, which are important sites of neurotransmitter opioid synthesis.

### Opioid mRNA expression

Prolonged inhibition of BP increases lasts for several days in patients with mild-to-moderate hypertension. In this respect, although limited in scope, studies have suggested that acupuncture may induce increased expression of mRNAs for opioid precursors in the brain for a period of 24–72 h [77, 78]. Recent data from our laboratory using real-time PCR demonstrated that the level of preproenkephalin in the rVLM is increased by 90 min after completion of a single 30-min application of EA at P5–6 acupoints in rats [79]. Moreover, conscious rats treated with repeated EA express higher levels of preproenkephalin for >24 h while the concentration of enkephalin in the rVLM is elevated for 48 h [80]. The roles of opioid mRNA expression and other neurotransmitter precursors, as well as the neurotransmitters released in the hypothalamus, midbrain, and other regions of the medulla, particularly in studies involving repetitive EA in sympathoexcitatory conditions, are worthy of further investigation.

### Endocrine and vascular actions of acupuncture

A previous study showed that 8 weeks of EA treatment at 350 Hz delayed development of hypertension and restored nitric oxide levels in the plasma of spontaneously hypertensive rats [81]. Additionally, prolonged intermittent stimulation for 2 h EA at S36 at 3 Hz induced nNOS expression in the gracile nucleus and medial nucleus tractus solitaries [82], suggesting that nNOS may participate in the effects of acupuncture on central cardiovascular regulation.

Acupuncture reduces BP through a modulation of the endocrine system that includes decreases in plasma renin, angiotensin II and aldosterone [14, 17, 23], and increases in the excretion of sodium [83]. Our recent clinical data show that EA weekly for 8 weeks decreased plasma levels of catecholamine, renin, and aldosterone in hypertensive patients who responded to EA treatment, but not in EA non-responders. Thus, these preliminary data suggest that the long-lasting EA-induced inhibition of hypertension is related to the inhibition of sympathetic activity, in particular, renal sympathetic nerves. The decreases in the release of renin, angiotensin, and aldosterone suggest an increase in the excretion of sodium and water that leads to a decrease in blood volume and reduced SBP. Additional studies are needed to confirm these findings.

### Limitations of this commentary

This article discusses over 23 articles of meta-analyses and clinical studies that largely provide inconclusive findings. The intention of this article is not to sum up all acupuncture studies, but to identify areas of concern and aspects that are important in determining the effectiveness of acupuncture to treat hypertension. We have not investigated a priori how EA alters the renin–angiotensin and aldosterone systems in our experimental investigation. Our preliminary clinical data indicate that EA alters plasma hormones after 8 weeks with weekly treatment at P5–6 + S36–37 in mild-to-moderate hypertensive patients. We also do not know if acupuncture alters parasympathetic activity in hypertensive subjects. Although our current ongoing clinical study showed that EA does not influence heart rate, we showed that EA increased parasympathetic activity during reflex responses. Moreover, in our clinical study, we only used P5–6 + S36–37 acupoints to treat hypertension. We have not examined other acupoints used by other acupuncturists in treating symptoms related to hypertension. Acupoints such as LI11, LI4, and other acupoints located on the head also project to the arcuate nucleus and may inhibit sympathetic activity and reduce hypertension. Furthermore, questions remain around how frequently patients should be treated to gain the maximum effect of acupuncture. Thus, we do not know if EA could reduce high BP significantly faster if applied twice or more per week to hypertensive patients. These issues require further study. Thus, although we have shown that EA modulates both sympathetic and parasympathetic systems in inhibitory cardiovascular responses, it is unclear if acupuncture also alters both nervous systems in hypertensive subjects.

## Summary and prospects

Low-current and low-frequency EA at P5–6 and S36–37 reduced high blood pressure in a subset of patients (about 70 %) with mild to moderate hypertension, and the effect showed a slow onset (4–8 weeks) but was long-lasting (1–2 months). EA inhibited cardiovascular sympathoexcitatory neurons through activation of neurons in the arcuate nucleus of the hypothalamus, the vlPAG in the midbrain and the NRP in the medulla, which in turn, inhibited the activity of premotor sympathetic neurons in the rVLM to reduce blood pressure. The arcuate also projects to the rVLM and contains endorphin. The neurotransmitters glutamate, acetylcholine, opioids, GABA, nociceptin, serotonin and endocannabinoids all participate in the EA hypotensive response, although their importance varies between nuclei. The long-lasting inhibition of EA is related to opioids and GABA in the rVLM, neural circuitry between the arcuate and vlPAG, and prolongation of the increase in preproenkephalin mRNA and enkephalin expression in the rVLM and arcuate. The inhibition of sympathetic activity, renin, angiotensin and aldosterone may also be quite important. Thus, a number of mechanisms underlying the long-lasting effect of EA on cardiovascular function have been suggested, but further investigation is warranted.

Acupuncture modulated low blood pressure and heart rate and EA influenced both sympathoinhibitory and parasympathoexcitatory reflex responses [84–87], suggesting that EA might restore the activity of the autonomic nervous system during suboptimal conditions. The spinal cord circuitry controlling the cardiovascular visceral reflex responses during EA requires further elucidation.

## Conclusion

The action of EA might be related to the activation of opioid system in the brain and inhibition of sympathetic activity and renin–angiotensin–aldosterone system. Future studies are required to improve our understanding of this mechanism.

## Abbreviations

BP: blood pressure
EA: electroacupuncture
rVLM: rostral ventrolateral medulla
vlPAG: ventrolateral periaqueductal gray
IML: intermediolateral column
